# Somatic genetic aberrations in benign breast disease and the risk of subsequent breast cancer

**DOI:** 10.1038/s41523-020-0165-z

**Published:** 2020-06-12

**Authors:** Zexian Zeng, Andy Vo, Xiaoyu Li, Ali Shidfar, Paulette Saldana, Luis Blanco, Xiaoling Xuei, Yuan Luo, Seema A. Khan, Susan E. Clare

**Affiliations:** 1 Division of Health and Biomedical Informatics, Department of Preventive Medicine, Northwestern University Feinberg School of Medicine, Chicago, IL USA; 2 Department of Data Sciences, Dana-Farber Cancer Institute, Harvard T. H. Chan School of Public Health, Boston, MA USA; 3 Committee on Developmental Biology and Regenerative Medicine, The University of Chicago, Chicago, IL USA; 4 Department of Medicine, Brigham and Women’s Hospital, Boston, MA USA; 5 Department of Surgery, Northwestern University Feinberg School of Medicine, Chicago, IL USA; 6 Department of Pathology, Northwestern University Feinberg School of Medicine, Chicago, IL USA; 7 Department of Medical and Molecular Genetics, Indiana University School of Medicine, Indianapolis, IN USA

**Keywords:** Breast cancer, Cancer genomics, Cancer prevention

## Abstract

It is largely unknown how the development of breast cancer (BC) is transduced by somatic genetic alterations in the benign breast. Since benign breast disease is an established risk factor for BC, we established a case-control study of women with a history of benign breast biopsy (BBB). Cases developed BC at least one year after BBB and controls did not develop BC over an average of 17 years following BBB. 135 cases were matched to 69 controls by age and type of benign change: non-proliferative or proliferation without atypia (PDWA). Whole-exome sequencing (WES) was performed for the BBB. Germline DNA (available from *n* = 26 participants) was utilized to develop a mutation-calling pipeline, to allow differentiation of somatic from germline variants. Among the 204 subjects, two known mutational signatures were identified, along with a currently uncatalogued signature that was significantly associated with triple negative BC (TNBC) (*p* = 0.007). The uncatalogued mutational signature was validated in 109 TNBCs from TCGA (*p* = 0.001). Compared to non-proliferative samples, PDWA harbors more abundant mutations at *PIK3CA* pH1047R (*p* < 0.001). Among the 26 BBB whose somatic copy number variation could be assessed, deletion of MLH3 is significantly associated with the mismatch repair mutational signature (*p* < 0.001). Matched BBB-cancer pairs were available for ten cases; several mutations were shared between BBB and cancers. This initial study of WES of BBB shows its potential for the identification of genetic alterations that portend breast oncogenesis. In future larger studies, robust personalized breast cancer risk indicators leading to novel interception paradigms can be assessed.

## Introduction

From 1989 to 2016 the mortality rate for breast cancer (BC) in the United States decreased by 40%^1^, a testament to the efficacy of targeted therapies, as well as to combinations and schedules of chemotherapeutics. During this same period breast cancer incidence rates remained static^1^; evidence of both the paucity of novel, effective prevention strategies that target specific molecular risk pathways, and our inability to implement existing strategies. Major barriers are two-fold: hesitation among healthy women to accept drugs for a disease that they may or may not experience in the future; and their reluctance to experience side effects that impair quality of life and may compromise health^2^. The first of these would be mitigated by improved identification of women at high risk of developing breast cancer, but almost 30 years after the initial publication of the Gail Model^3^, breast cancer risk stratification remains imprecise and insensitive to breast cancer subtype. In an analysis of data from the Women’s Health Initiative, the Gail Model displayed modest ability to predict the risk of breast cancer (AUC = 0.58, 95% CI = 0.56–0.60)^4^. Among women at high risk of breast cancer, for example, those diagnosed with atypical hyperplasia, neither the Gail Model/Breast Cancer Risk Assessment Tool nor the Tyrer-Cuzick Model performed well^5,6^. This is a significant barrier to implementation of established medical interventions for disease prevention, and to the development of new, targeted intervention strategies for women at risk. Impactful, targeted prevention strategies require knowledge of how breast cancer risk is transduced at the molecular level in the breast itself, i.e., identification of somatic genetic changes that predate breast cancer and influence the biologic profile of cancers that emerge.

Benign breast disease is an established risk factor for BC^7,8^, with 30% of BC cases reporting a history of benign breast disease^9^. Of the 1.7 million breast biopsies each year in the U.S.^10^, about 75% of these return a diagnosis of benign breast disease, including atypical hyperplasia^9^. This provides a window into the somatic genetic environment of the breast, prompting us to evaluate the genetic landscape of benign breast biopsy (BBB), and identify patterns associated with subsequent malignancy. Starting in the embryo^11^, tissues accumulate DNA mutations over time^12^. Most of the mutations are repaired, many are inconsequential, but a few may lead to cancer^13,14^. Before there is any histologic evidence of invasive cancer, histologically normal, and benign tissue contain molecular aberrations that are associated with malignancy^15,16^. For example, sun-exposed, normal eyelid skin has been shown to have a mutation burden of 2–6 mutations/MB/cell, a rate similar to that observed in many cancers^17^. The processes that cause these mutations leave an imprint on the genome^18^. In the sun-exposed eyelid epidermis, mutations occur within a pattern that mimics the Welcome Trust Sanger Institute (WTSI) Mutation Signature, which is associated with ultraviolet exposure and its consequent CC > TT dinucleotide mutations at dipyrimidines^19^. Exogenous or endogenous mutational processes, such as that which produced WTSI signature 7, are chemical reactions within DNA. While mutational processes are responsible for the creation of mutations, the mutations that are observed ultimately within a malignancy reflect a process of selection^20^. However, genetic aberrations are not limited to somatic mutations, and we note that recurrent copy number variations (CNVs) are in fact more characteristic of invasive breast cancers than recurrent mutations^20^.

To evaluate the molecular alterations that enable cancer development in the breast, we established a case-control study of BBB samples, the Benign Breast & Cancer Risk (BBCAR) Study. We performed whole-exome sequencing (WES) on the benign biopsies of patients, who subsequently developed breast cancer (cases), and matched controls, who have not developed breast cancer to date. The cases and controls had similar degrees of benign change: non-proliferative or proliferation without atypia. The focus on non-atypical lesions was a deliberate choice as non-atypical lesions predict a generalized risk of subsequent breast cancer, occurring equally frequently in both breasts^9^. They are also far more common than atypical changes, comprising over 90% of all breast biopsies^21^, so that elucidation of their molecular profiles will impact the majority of women who undergo BBB. To the best of our knowledge, WES has not been performed in any previous case-control study of benign breast lesions without atypia. In addition to profiling the overall BBB mutational landscape, we have determined that mutations differ as a function of the type of benign breast disease, specifically that the *PIK3CA* pH1047R hotspot mutation is more frequent in proliferative disease without atypia (PDWA) compared to non-proliferative disease (*p* < 0.001); our data reveals a presently uncatalogued mutational signature associated with TNBC (*p* = 0.007), which was validated in 109 TCGA TNBC samples (*p* = 0.001); and we observed multiple recurrent CNVs, including a MLH3 deletion, which is significantly associated with a mismatch repair signature (*p* < 0.001).

## Results

### Study design

**Fig. 1:**
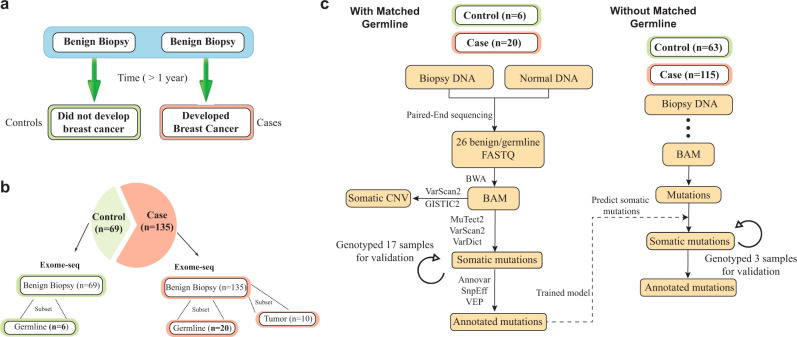
Case-control study of benign breast biopsy (BBB) samples. **a** Design of the BBCAR study. The sample tissues of subjects who subsequently developed breast cancer (cases), and their matched controls, who have not developed breast cancer to date, were studied **b** A total of 135 cases, matched to 69 controls, were selected for whole-exome sequencing (WES). Case and control samples are matched by age and histology **c**. an illustration of the workflow to identify somatic mutations in the tissue samples that lack matched germline DNA. To train a model to distinguish germline variants and somatic mutations, previously consented donors were re-contacted (with IRB approval) and saliva specimens were requested for germline DNA sequencing. Orthogonal SNP array genotyping was further performed with 20 samples to compare and validate the performance of somatic mutation identification.

A total of 204 subjects were enrolled in this BBB case-control study. Cases (*n* = 135) are women who have undergone a breast biopsy with specimen histology showing non-proliferative disease or proliferative disease without atypia (Supplementary Fig. 1) that predates the diagnosis of breast cancer by at least one year (Fig. 1a). The median interval from benign biopsy to the diagnosis of cancer is 7.3 (SD = 4.4) years. Controls (*n* = 69) are women who have not developed breast cancer and are matched for age of diagnosis (±2 years) and histology (Fig. 1b; Table 1). Controls were verified to not have been diagnosed with breast cancer as of 08/14/2018 (Supplementary Data 1)^22^.

**Table 1:** Distributions of demographic data and tumor characteristics between the Case group and the Control group Student’s *t*-tests were performed for continuous variables and Pearson’s Chi-squared tests were performed for categorical variables.

	Case (135)	Control (69)	*P*-value
Age (SD) Mean (SD)	49.7 (9.9)	49.8 (9.6)	0.96
Menopausal status *N* (%)			0.87
Pre 114	76 (56.3%)	38 (55.1%)	
Post 90	59 (43.7%)	31 (44.9%)	
Histology Class *N (%)*			0.78
^ a^Class 1 115	79 (58.5%)	40 (58.0%)	
51 (37.8%)	25 (36.2%)	^ b^Class 2 75	
	NA 10	5 (3.7%)	4 (5.8%)
ER status *N (%)*			
Positive	109 (80.8%)		
Negative	23 (17.0%)		
Low	3 (2.2%)		
Follow-up years (SD)	7.3 (4.4)	16.6 (5.4)	<0.01
Has matched germline (%)	20 (14.8%)	6 (8.7%)	
Has matched cancer (%)	10 (7.4%)		

^a^Class 1/non-proliferative: “Non-proliferation” and “Benign, NOS”.

^b^Class 2/proliferative: “Proliferative lesion without atypia” (includes non-atypical hyperplasia, radial scar, sclerosing adenosis).

### Somatic mutation identification

**Fig. 2:**
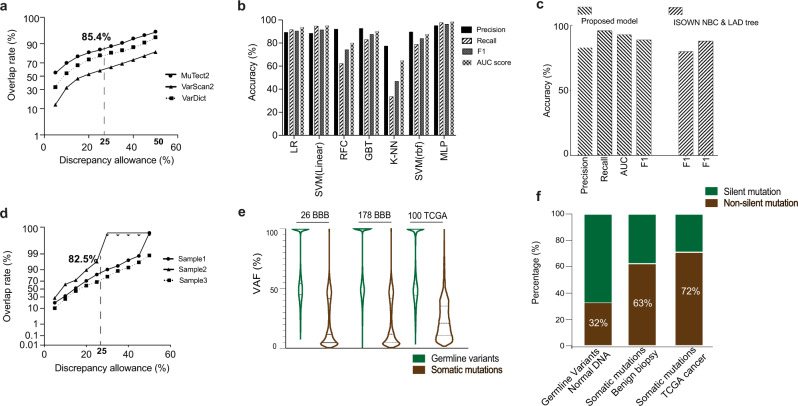
Accurate somatic mutation identification in benign biopsies. **a** Comparison of the mutations between WES and genotype array. Somatic mutations were called using Mutect2, VarScan2, and VarDict. With different allele frequency discrepancy allowance, the overlap rate between two platforms was plotted. **b** Performance of different machine learning models in the test set. Penalized logistic regression (LR); linear SVM; random forest classifier (RFC); gradient boosted tree (GBT); k-nearest neighbor algorithm (K-NN); SVM with rbf kernel; multiple layer perceptron (MLP). **c** Orthogonal validation of the proposed model using 100 TCGA breast cancer samples and benchmark study with previously validated pipelines, including ISOWN NBC and ISONWN LAD tree^26^. **d** Pipeline validation using genotype arrays of three samples. Somatic mutations were called using our pipeline and validated by genotyping. The plot shows the overlap rate between the two platforms with different allele frequency discrepancy allowance. **e** VAF distribution of the germline variants and somatic mutations, grouped by 26 benign biopsies with matched normal DNA, 178 benign biopsies lacking matched normal DNA, and 100 randomly selected TCGA breast cancer samples. **f** Distribution of silent and non-silent mutations, grouped by germline variants called in normal DNA that matched to the BBB, somatic mutations in BBB, and somatic mutations in TCGA breast cancer samples.

All 204 specimens were dissected using laser capture microdissection (LCM) and were subjected to WES^23^. Within this cohort, 26 matched germline DNA were obtained for WES as well. To evaluate mutation caller performance in this benign tissue setting, 17 of the 26 sample pairs were subjected to genotyping in order to evaluate mutation caller performance (Fig. 1c; “Methods”, “Supplementary Materials and Methods”). Allele frequencies of the mutations common to the genotyping array and WES were compared. Mutations were categorized as false positive if allele frequency was discrepant between the two platforms. The mutation identification accuracy then varies as a function of the discrepancy allowance (Fig. 2a). Overall, we observed high consistency between the two platforms (85.4% when discrepancy allowance = 25%). Notably, MuTect2 consistently achieved better performances in this setting (Fig. 2a), Therefore, MuTect2 was selected as mutation caller for subsequent studies.

For the samples lacking matched normal DNA (*n* = 178), a machine learning model was developed to distinguish germline variants and somatic mutations (Fig. 1c; “Methods”; “Supplementary Materials and Methods”). With somatic mutations called for the 26 samples for which germline DNA was available, we systematically evaluated multiple machine learning approaches to distinguish somatic mutations and germline variants in benign biopsies (Fig. 1c; “Methods”; “Supplementary Materials and Methods”). A total of 31 features were utilized for the model evaluation (Supplementary Table 1), including protein structure, pathogenicity prediction, population frequency, or evolutionary factors^24^. Various functional annotation or toxicity scores were derived from ANNOVAR^25^, COSMIC (https://cancer.sanger.ac.uk/cosmic), dbSNP/common (https://www.ncbi.nlm.nih.gov), along with intrinsic sequencing features, such as mutation allele frequency, depth of reference reads, mutation frequency among the cohort. Grid search was applied to unbiasedly tune each model’s parameters using five-fold cross-validation on the training set. Evaluation performance was then achieved on the held-out test set (“Methods”). Of the evaluated models including penalized logistic regression (LR), linear SVM, random forest classifier (RFC), gradient boosted tree (GBT), k-nearest neighbor algorithm (K-NN), SVM with rbf kernel, and multi-layer perceptron (MLP), MLP model achieved the best performance (Fig. 2b), where the F1-score is 0.96 (Supplementary Table 2).

Orthogonal validations of the proposed model were performed by evaluation studies with the TCGA data and benchmark studies with previously validated pipelines. Protected datasets in bam format of 100 randomly selected breast primary tumors were downloaded directly from the TCGA data portal. Realigned raw reads were subjected to base recalibration and were passed to Mutect2 for mutation detection. Mutect2 was performed in so called “tumor only mode” to call somatic and germline mutations. ISOWN^26^, a previously validated pipeline for somatic mutation identification, was applied for somatic mutation prediction as well. The predicted results were evaluated by comparison to the TCGA somatic mutation data by Multi-Center Mutation-Calling in Multiple Cancers (MC3 public v0.2.8) network^27^. Using the TCGA MC3 data as ground truth, our model achieved a F1-score of 0.89 (Fig. 2c) in predicting somatic mutations. Even though designed and trained in the benign-biopsy setting, our model (F1 = 0.89) obtained similar or better results than previously validated pipelines, such as ISOWN NBC (F1 = 0.88) and ISOWN LAD tree^26^ (F1 = 0.80) in predicting somatic mutations in TCGA cancer samples (Fig. 2c).

We further applied our pipeline and model to identify somatic mutations in the 178 BBBs lacking matched normal DNA (Fig. 1c) (Methods). Overall, the average read depth for the identified somatic mutations is 99, whereas the average VAF is 0.232. To estimate the overall mutation identification accuracy, we randomly sampled and genotyped three samples from our cohort. Overall, we observed high consistency between our pipeline and the genotype array (82.5% when discrepancy allowance = 25%) (Fig. 2d). As a sanity check, the distribution of variant allele frequency (VAF) and non-silent mutations were examined. Consistent with previously reported studies^28,29^, the majority of our identified germline variants’ VAFs are around 50% and 100%, whereas somatic mutations display much lower VAFs (Fig. 2e). For cancers, non-silent mutations usually account for 2/3 of somatic mutations with the remaining 1/3 being silent mutations, whereas germline mutations are expected to have higher number of silent mutations^28^. In our data, we have observed similar distribution (TCGA 100 breast cancer: 72% non-silent mutations) (Fig. 2f). In addition, we observed an increasing spectrum of non-silent mutations in BBB matched normal DNA (32%), BBB (63%), and TCGA cancer samples (72%) (Fig. 2f). To note, the average non-synonymous mutations for the 26 BBBs with matched normal DNA is 114, whereas the average number for the 178 BBBs without matched normal DNA is 127.

### Mutation catalogues

**Fig. 3:**
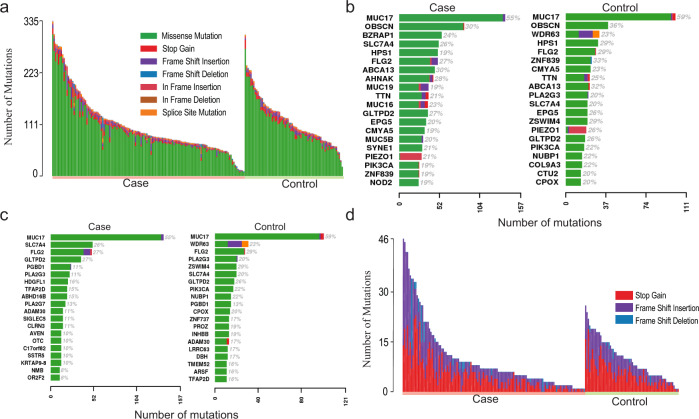
Catalog of somatic mutations in 204 benign breast biopsies. **a** Catalog of base substitutions, insertions/deletions in the 204 BBBs. Each bar represents one individual’s total number of mutations. Left panel is the case and right panel is the control. **b** The top 20 mutated genes in the case group (left) and control group (right). **c** The top mutated genes as b, adjusted by gene length. **d** Catalog of nonsense mutations in the 204 BBBs.

Among the 204 samples, 36,801 somatic base substitutions and 2283 small INDELs were identified. The majority of the mutations were missense mutations (Fig. 3a). Cases had a mean of 6.2 mutations/MB (SD = 3.6) and controls had 6.8 mutations/MB (SD = 3.0). No significant difference was observed in the numbers of mutations between the cases and controls (Fig. 3a). Among the top 20 mutated genes, the case group and control group shared common genes (*MUC17*, *OBSCN*, *FLG2*, *GLTPD2*, *ABCA13, PIK3CA*) (Fig. 3b). Approximately one-fifth of both cases and controls display *PIK3CA* mutations, with the highest frequency at pH1047R. When corrected by gene length, case and control still shared common genes (*MUC17, SLC7A4, FLG2, GLTPD2, PGBD1, PLA2G3, ADAM30*) (Fig. 3c). Mucins are O-glycosylated by the addition of N-acetylgalactosamine to the hydroxyl group of serine or threonine^30^. Therefore, we evaluated the number of missense mutations within MUC17 that resulted in the gain or loss of either serine or threonine residues. Of the MUC17 mutations we observed, 8.7% of missense mutations would be predicted to result in the loss of serine, 16.8% in the loss of threonine, 14.2% in the gain of serine and 17.8% in the gain of threonine. However, there was no significant difference between cases and controls (Supplementary Table 3). The proportions of nonsense mutations also vary between samples. The majority of nonsense mutations were frame shift insertions and stop gains, with some exceptions in a few samples (Fig. 3d).

### Genes enriched for mutations in the cases or PDWA

**Fig. 4:**
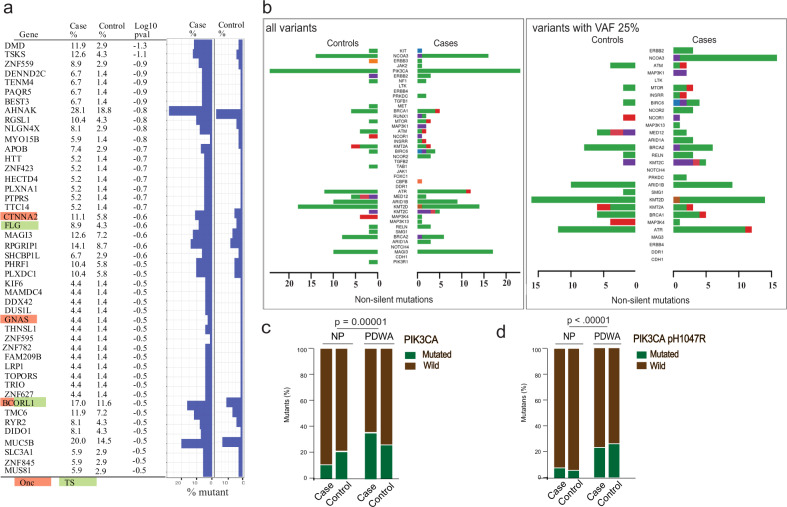
Genetic aberrations that distinguish case/control or proliferative/non-proliferative BBB. **a** For each gene, the percentage of mutated lesions in the case and control are shown. In the left panel, known oncogenes are highlighted as green, and known tumor suppressor genes are highlighted as orange. Onc is a known oncogene; TS is a tumor suppressor gene (Cancer Gene Census; https://cancer.sanger.ac.uk/census). The right panel shows the nonsynonymous rate in each group. **b** Mutations in 83 selected genes presented by Rohan et al.^31^ to validate our data and also to facilitate comparisons. Left: all non-silent mutations; right: non-silent mutations with VAF > 25%. **c** PIK3CA non-synonymous mutations identified in NP BBB and PDWA BBB. **d** Same as **c**, with only mutations at PIK3CA pH1047R retained.

To determine the enrichment of mutations in the case group, a logistic regression model was fit for each gene, with case/control as output variable and mutation status as input variables. The *p*-values were derived from the fitted models for gene sorting (Fig. 4a). Nonsynonymous mutations in four cancer-associated genes, *CTNNA2* (11.1% vs. 5.8%; log10 *p*-value = −0.6), *FLG* (8.9% vs. 4.3%; log10 *p*-value = −0.6), *GNAS* (4.4% vs. 1.4%; log10 *p*-value = −0.5), and *BCORL1* (17.0% vs. 11.6%; log10 *p*-value = −0.5*)*, were more abundant in the case group. Of note, same analyses including synonymous mutations are presented in Supplementary Fig. 2.

Rohan and colleagues utilized targeted sequence capture to identify mutations present in a panel of 83 genes in the benign breast disease tissue from a case-control study^31^. While they identified somatic mutations in a number of genes frequently mutated in breast cancer, no significant differences were identified comparing cases and controls with regard to the mutational burden, genes mutated, type of mutation or pathway. We queried our data for the mutations present in these same 83 genes. Our data for all variants was very similar to theirs (Fig. 4b), which orthogonally validated our data quality. Nonetheless, differences were observed after filtering for variants with a VAF > 25%; in particular, while no variants in *NCOA3* had a VAF greater than 25% in the controls, over 10% of cases passed this threshold (Fig. 4b).

We also evaluated mutation enrichments in benign biopsies showing proliferative disease without atypia (PDWA) (*n* = 76) versus non-proliferate (NP) disease (*n* = 119). Using non-synonymous mutations only, the top enriched significant genes are *PIK3CA, HYDIN*, *DNMT3B*, and *AKT1* (detail of hotspots in Supplementary data 2)^22^. For *PIK3CA*, mutations are abundantly enriched in PDWA compared to NP (31% vs. 12%; *p* = 0.00001) (Fig. 4c). Specifically, pH1047R is the most enriched hotspot for the PDWA (28% vs. 5%; *p* < 0.00001) (Fig. 4d)

### Mutational processes and CNV

**Fig. 5:**
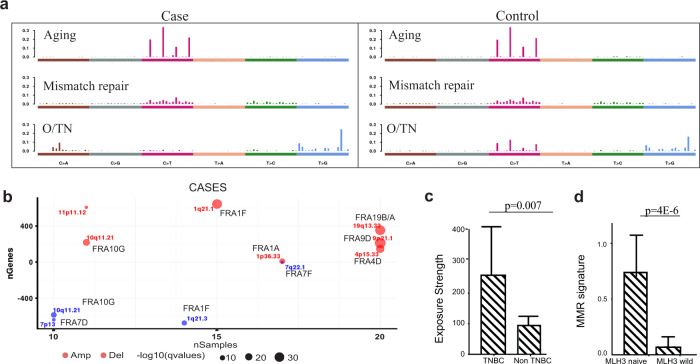
Mutational processes and somatic copy number variation (CNV) identified in the case and control groups. **a** The identified mutation signatures were compared with those of the Welcome Trust Sanger Institute. The aging signature and mismatch repair signature (MMR) are enriched in both groups. The uncatalogued signature O/TN is enriched with T > G/A > C mutations, with 5′GAA3′ > 5′GCA3′ the most frequently mutated trinucleotide. **b** Recurrent somatic copy number variation in the case group. Common fragile sites are labeled. The size of the dots represents the q-value (FDR adjusted *p*-value). Red are amplifications, blue are deletions. *y*-axis is the number of genes involved, and *x*-axis is the number of samples involved. **c** The uncatalogued signature is enriched in BBBs that predate triple negative breast cancer. Each sample is assigned a continuous number representing the signature exposure strength, which was the product of matrix decomposition. **d** The mismatch repair signature is highly abundant in the BBB with MLH3 deletion (MLH3 naive). Error bars are 95% confidence interval (CI).

Mutations are non-random and occur within sequence motifs. These motifs provide evidence from which we can infer the process that created the mutations. Recent studies led by investigators at the Welcome Trust Sanger Institute (WTSI) present the somatic mutation data as a 96-element vector, which captures the immediate 5′ and 3′ neighbors of the mutated nucleotides. Employing non-negative matrix factorization (NMF), 30 “mutational signatures” were produced by these studies^19,32^, which more recently has been updated and expanded to 40^33^. We hypothesized that like the eyelid epidermis^17^, benign breast lesions also harbor somatic mutations with associated mutational signatures that may provide clues to etiologic processes. Within the BBB cohort, mutational signatures were examined. Three mutational signatures were identified in both case and control group (Supplementary data 3)^22^. In both groups, we identified the “aging” signature (cataloged by WTSI as Signature 1b; Fig. 5a; cosine similarity score: 83.2% for the case and 83.0% for the control), which is the putative result of the hydrolysis 5-methylcytosine. We also identified the “mismatch repair” signature (cataloged by WTSI as Signature 6; Fig. 5a; cosine similarity score: 80.5% for the case and 80.1% for the control). Moreover, a signature not currently in the WTSI catalog of Mutational Signatures was identified in each group; both demonstrate enrichment of T > G mutations with 5′TTC3′ > 5′TGC3′ the most frequently mutated trinucleotide motif (Fig. 5a). Provisionally, we have named this signature “O/TN” based on the presumed mechanism: oxidation, and on its presumptive association with triple negative (TN) breast cancer.

The process of deriving mutational signatures is an unsupervised learning process. Pooling the cases and controls, we derived three signatures in the BBB cohort, namely aging, mismatch repair, and O/TN. In an association study, we found that O/TN was significantly associated with BBB that predate TNBC (*p* = 0.007) (Fig. 5c). We also performed a second association analysis, controlling for the potential covariates of age, menopausal status, and histology class (NP or PDWA). The association remained significant (*p* = 0.016), suggesting that the O/TN signature in BBB is predictive of TNBC. To validate the O/TN signature and examine whether it is a predictor of TNBC as well, we further retrieved 109 TNBC samples from TCGA data portal. The downloaded somatic mutation data were processed, and three mutational signatures were derived under the same protocol as BBB (Methods). As result, we were able to identify the O/TN signature in the TCGA TNBC cohort (Cosine = 0.72, *p* = 0.001).

A majority of breast tumors, especially those that are HER2 positive, have been reported to be enriched with mutations hypothesized to result from the action of the APOBEC enzymes^34^. In our cohort, no tumors were found to be enriched with mutations within the APOBEC motif, nor did we observe either WTSI Signatures 2 or 13, both of which are hypothesized to be the result of the activity of these enzymes. We have also examined the subset of 11 BBB that eventually developed HER2 positive cancer and the subset of 29 BBB that developed cancer within 3 years of biopsy, and we found no APOBEC signatures enriched in these BBB.

We also employed VarScan2^35^ to study somatic CNV in the 26 samples for which we have matched normal DNA. The learnt segments were then passed to GISTIC2^36^ for recurrent CNVs study (genome-wide CNV variation: Supplementary Fig. 3). We observed that majority of the cytobands occur at or immediately next to common fragile sites, suggesting these cells are under considerable replication stress (Fig. 5b). The observed cytobands at which CNVs map exclusively in the cases have been associated with cancers, in general or invasive breast cancers, in particular. Amplifications are hypothesized to be the result of breakage-fusion-bridge (BFB) cycles triggered at the induction of fragile sites^37^. One of the amplifications identified in the BBCAR cases is an amplification outlier identified using breast cancers from the METABRIC consortium that mapped to chr19q13.33, which contains 26 genes. No candidate oncogene has yet to be identified within this amplicon^38^. Chromosome 1q21 is the fourth most frequent locus of copy number variations in cancer^39^.

To investigate the mechanisms underlying our mismatch repair signature, mismatch repair genes *MLH1, MLH3, MSH2, MSH3, MSH6, PMS2, MUTYH, MYH11, SETD2 and TGFBR* were examined for deleterious mutations and/or deletion in the subset of samples with matched germline DNA available (*N* = 26)^40^. Approximately one-third of the cases and controls have at least one mutation in one of the mismatch repair associated genes (Supplementary data 4)^22^. *PMS2* is deleted in one-half of the cases (10/20) and *MLH3* in all of the controls (6/6). However, only one of the 10 cases displaying a *PMS2* deletion also evidenced a mutation in an MMR associated gene, specifically *SETD2*. None of the controls with MLH3 deletions carried a mutation in any of the MMR associated genes. Strikingly, benign biopsies harboring a MLH3 deletion are abundantly enriched with the mismatch repair signature compared to MLH3 wild biopsies (*p* = 4.2E-6) (Fig. 5d).

### Cancer risk prediction at BBB

In an attempt to build a model for cancer prediction at the time of BBB using somatic information, we fit logistic regression with L1 penalty using the case/control status as output variable. To reduce the number of input prediction features, all somatic mutations that were annotated with same protein domain were aggregated as a continuous number, representing the mutation burden of the corresponding protein domain. In total, 1966 annotated protein domains were utilized as input features for case/control prediction (Supplementary data 5)^22^. To evaluate the model and features, we performed a bootstrapping by randomly splitting the BBB samples at a 7:3 ratio, and trained the model using 70% of the samples, in which 30% of the samples were used as test set. We repeated the process ten times and obtained an AUC for each run. As a result, we obtained an AUC score of 67% (95% CI = 63.1–70.9%) in predicting the cases. Of note, the inclusion of clinical characteristics and demographics, including age at the time of BBB, age at menarche, age at first live birth, family history of breast cancer in a first-degree relative, histologic variable (proliferative vs non-proliferative), did not improve the model’s performance.

### Somatic mutations present in both benign biopsy and cancer

Our cases were defined as BBB that predate breast cancer. In this study, to longitudinally compare mutations in the BBB and in the cancer samples, we retrieved ten tumors that matched to our BBB cohort. Preprocessing for mutation calling was performed as for the BBB, including laser capture microdissection (LCM), DNA extraction, library construction, sequencing, alignment, mutation calling, and variant filtering. Of the identified mutations in these ten cancer samples, 957 were observed in both the benign biopsies and cancer tissues (Supplementary data 6)^22^. The average allele frequencies for these mutations is 32.2% (SD = 18.7%) in the BBB and 46.7% (SD = 17.3%) in the cancer tissues. *FAT1*, *CTNNA2*, *ATR and ETAA1* were among the top ten mutated genes (Supplementary Table 4); these are known tumor suppressor genes or oncogenes. All six of the *CTNNA2* mutations occur within the motif 5′GAA3′ > 5′GCA3′. This motif is a predominant feature of our O/TN mutation signature (Fig. 3).

## Discussion

Genetic aberrations associated with malignancy occur within normal tissues^17^ and within tissues at the population risk of breast cancer^15^ as well as within lesions at substantial risk^16^. A previous case-control study performed by Rohan et al., with a design that closely mirrors ours, utilized targeted sequence capture^31^; no significant differences between cases and controls with regard to somatic mutations were identified and no mutations were shared between the biopsy and tumor pairs. Comparing the number of somatic mutations identified in their targeted genes with these same genes in our WES data revealed striking similarity and to make the similarity easy to discern, we designed Fig. 4b to mirror their Fig. 1a, b. Soysal and colleagues also employed targeted sequencing in an attempt to identify somatic mutations present in antecedent fibrocystic disease (FD) and subsequent invasive breast cancers^41^. In contrast to our study and that of Rohan et al.^31^, no significant somatic mutations were identified in the FD. In their discussion section Rohan et al. suggested that “more detailed approaches (e.g., exome/whole-genome sequencing)” might prove more informative than targeted sequencing^31^. We employed WES in a similar case-control setting. We rigorously evaluated the sequencing quality, mutation calling, and mutation classification. Since we did not have germline samples available from most of our subjects, we developed a neural network model to predict somatic mutations for the benign biopsies, which we were able to accomplish with a F1 score of 96%. This tool was further validated in TCGA (MC3) data with a F1 score of 89%. Using the sequencing data produced, we have identified recurrent mutated genes. We also built a predictive model for the risk of breast cancer using genetic information alone and obtained an AUC of 67% (95% CI = 63.1–70.9%). This represents the best performance to date using benign breast lesions, despite the exclusion of subjects with atypical hyperplasia^42^. Importantly, we have identified a currently uncatalogued signature, which we have designated O/TN, that is associated with triple negative breast cancer (*p* = 0.007), which was validated in 109 TCGA TNBC samples (*p* = 0.001); we found that *PIK3CA* pH1047R hotspot mutation is more frequent in proliferative disease without atypia (PDWA) compared to non-proliferative disease (*p* < 0.001); we observed multiple recurrent CNVs as well, including a MLH3 deletion, which is significantly associated with a mismatch repair signature (*p* < 0.001).

This study has several strengths and weaknesses. The specimens are richly annotated with clinical information (Supplementary Data 1)^22^ and they have been laboriously microdissected in order to sample the epithelial compartment. The controls have a long median follow up and were verified not to have been diagnosed with BC by a telephone interview carried out at the time of this study. We have leveraged the advantages of machine learning/artificial intelligence to enable the calling of somatic mutations in the absence of germline data.

Weaknesses include the relatively small size of the study and the lack of an independent validation dataset, so that the findings we report here must be regarded as preliminary until larger numbers can be studied. We were able to obtain germline specimens on only 26 subjects. Data from these 26 specimens was utilized to build the Panel of Normals (PoN) for germline variant filtering; GATK recommends a minimum of 40. Using less than the suggested minimum may result in suboptimal denoising of the data and may not capture all the common germline variants. Since all subjects consented to participation and to recontact, we are working actively to acquire additional germline samples. Finally, the use of formalin-fixed paraffin embedded breast samples, although unavoidable in this setting, risks introducing artefactual findings. Among the FFPE artifacts are C to T transitions hypothesized to be the result of the deamination of cytosines^43,44^ and both Rohan et al.^31^ and Soyal et al.^41^ screened for these substitutions. However, a large, prospective study carried out by the 100,000 Genomes Project argues that the choice of mutation caller as well as tissue heterogeneity or sampling may contribute to differences between FFPE and frozen tissue^45^. Our O/TN signature is not dominated by C to T transitions. Although our MMR signature and aging signatures are populated by C to T transitions, we think it unlikely that these are due to formalin-induced deamination, as our signatures closely mirror those of WTSI 6 and 1b, respectively, which were derived from frozen specimens. Considering the risk of ruling out true mutations, therefore, we did not attempt to account the FFPE artifacts in our pipeline.

In contrast to our findings, Soysal et. al found no specific mutations in their study of benign breast lesions. Their depth of sequencing was more than adequate but only 17 patient samples were included^41^, with lesions that they called “fibrocystic breast disease with or without UDH, FEA, or CCL”. These lesions, with the exception of flat epithelial atypia, were also included in our study, making it unlikely that the choice of histology is determinative. We should consider the possibility that single nucleotide somatic mutation is not the correct genetic determinant of risk. Single cell sequencing of synchronous DCIS and invasive ductal carcinomas has revealed that CNV is early oncogenic event, i.e., present in in situ lesions, and that no additional CNV events are acquired during the transition from in situ to invasive lesion^46^. In a study separate from the one referred to earlier, Soysal et al. showed that *ESR1* gene amplifications are an early event in breast carcinogenesis and are already present, at least in part, in FD^47^. Additionally, recurrent CNVs are more characteristic of invasive breast cancers than are recurrent mutations^39^. Key breast cancer phenotypes, including intrinsic molecular subtypes, estrogen receptor status, and *TP53* mutation status as well as proliferative status and estrogen-signaling pathway activity can be predicted by DNA copy number features alone^48^.

Lesions such as hyperplasia, not all of which are obligate precursors of malignancy, already show evidence of activation of DNA damage response pathways. This is a response to oncogene-induced DNA replication stress causing unscheduled S-phase entry with consequent aberrant replication structures and DNA damage, which activate ATR/Chk1, ATM/Chk2, and p53, ultimately preventing progression by arresting growth or triggering cell death^49^. Intriguingly, with regard to our data, is the fact that in the early lesions that are genetically most stable, loss of heterozygosity at known fragile sites is observed to occur 3–15 times more often than expected from random targeting of these sites^49^. Fragile sites were also noted to be targeted during the period in which DNA damage response is maximal. These data suggest a model in which oncogene activation is an early event in at-risk tissue and that cells activate the ATR/ATM-regulated DNA damage responses that delay or prevent malignant progression. This may explain why we observe equivalent somatic mutations, e.g. *PIK3CA* (H1047R), in cases and controls. The *ATR* and *ETAA1* mutations that we observed in the BBCAR specimens and their matched tumors may be the specific mutations that enable oncogenic progression in the cases. Inactivating mutations including any in the ATM/Chk2 or ATR/Chk1 pathways potentially would remove the barrier to progression and result in cell proliferation and survival, increasing genomic instability and tumor progression.

Our O/TN signature is enriched with T > G/A > C mutations, with 5′TTC3′ > 5′TGC3′ the most frequently mutated trinucleotide motif. These single nucleotide T > G transversions are observed in vitro when equimolar oxidized dGTP (8-O-dGTP) is included in the nucleotide pool^50^. Strand information is lost between the initial occurrence of a mutagenic lesion and the ultimate readout by DNA sequencing. Conventionally, mutational signatures are displayed with a mutated pyrimidine at the center of the trinucleotide motif. The complement to 5′TTC3′ > 5′TGC3′ is 5′GAA3′ > 5′GCA3′. There is a 4- to 5-fold difference in the 8-O-dGTP mutation rate depending on the sequence context with 5′GAA3′ being a favored context^50^. The nucleotide pool is sanitized by MTH1, which hydrolyzes cytotoxic oxidized dNTPs, preventing them from becoming mis-incorporated into DNA during replication or repair. Even with this cellular sanitizing activity, nucleotide pools contain enough 8-oxo-dGTP to promote mutagenesis^51,52^. Mutagenesis results from the insertion of 8-O-dGTP across from adenine rather than cytosine during DNA replication. Steric hindrance of the oxygen of cytosine (C) in the anti-conformation with the triphosphate group of the 8-oxo-dGTP also in the anti-conformation prevents Watson-Crick base pairing^53^. However, 8-oxo-dGTP can assume the syn conformation enabling Hoogsteen base pairing with Adenine (A). This A(template): G^ox^ (nascent) base paring results in T > G/A > C following two additional rounds of replication^53^.

The association of triple negative breast cancer (TNBC) with our O/TN signature is intriguing. About 80% of TNBCs are of the basal-like subtype^54^ and this subtype likely originates from luminal progenitor cells^55,56^. We hypothesize that the O/TN signature results from deficient repair of a specific oxidative lesion as discussed above. The levels of reactive oxygen species and antioxidant defenses have been assayed in both luminal progenitor (LP) and basal cells of normal human mammary tissue^57^. Higher levels of both superoxide anion and hydrogen peroxide are present in the LPs. Even though multiple antioxidants are deployed, LP display higher levels of oxidative damage, specifically increased incorporation of 8-oxo-deoxyguanosine (8-oxo-dG) within the genomic DNA. Therefore, the association of TNBC with our O/TN signature may reflect the susceptibility of its precursor LP cells to oxidative damage, placing them at a disadvantage if this damage cannot be adequately addressed due to mismatch deficiency.

MHL3 deletion was strongly associated with our MMR signature. The trinucleotide motifs most frequently mutated in our MMR signature and WTSI Signature 6 are 5′GCG3′ > 5′GTG3′, 5′CCG3′ > 5′CTG3′, 5′ACG3′ > 5′ATG3′. These mutations are hypothesized to due to an error in the replication of 5-methylcytosine (5mC). Tomkomva et al. have advanced a model, which posits that wildtype Pol ε has slightly decreased fidelity when encountering 5mC, particularly in a GCG context, on the template strand and incorrectly pairs it with A, leading to 5mC:A mismatches^58^. They note that there is high structural similarity between 5mC and T, both of which present a methyl group at the same position of pyrimidine ring. If the resulting 5mC:A mismatches are not repaired before the next round of replication due to dysfunctional mismatch repair, one would expect an enrichment of NCG > NTG mutations. Given this hypothesized etiology of the mutations, is there evidence that MHL3 repairs such mutations? Sequencing of the tumors arising from the cross of *Apc1638N* mutant mice with *Mhl3* nullizygous and *Mlh3*^-/-^; *Pms2*^-/-^ mice reveals that the C:G > T:A transition mutations, irrespective of MMR genotypes, occurred at either CpG dinucleotides or CpNpG sites, typical targets for DNA methylation^59^. Thus, although our numbers are small, it appears that our MMR Signature in the controls may result from *MLH3* deletion; as the same signature is observed in the cases another mechanism of MLH3 silencing may be operative in the cases such as promoter methylation.

The ten most frequently mutated genes shared between the BBB of cases and their tumors are given in Supplementary Table 4. Among them are *FAT1*, *CTNNA2*, *ATR*, and *ETAA1*. *FAT1* has the most mutations, which is interesting as this same gene was shown to have a statistically significant excess of inactivating mutations across all classes in the sun-exposed, physiologically normal epidermis study^17^. *FAT1* encodes a cadherin-like protein and its inactivation via mutation may lead to tumorigenesis by multiple avenues^60,61^. From a breast cancer standpoint, investigations into the etiology of CDK4/6 inhibitor resistance have provided significant clues to FAT1’s role as a tumor suppressor^62^. Loss of FAT1 activity results in increased expression of CDK6, consequent to dysregulation of the Hippo pathway. *ATR* and *ETAA1* have been discussed earlier regarding their function as barriers to progression. We hypothesize that the CNV we have observed is due to replication stress. Replication stress leads to stalled replication forks and if *ATR* or *ETAA1* mutation renders the proteins unable to stabilize the forks and allow time for repair, further genomic instability in the genome is likely to ensue^63^. ATR also specially regulates fragile site stability^64^. While admittedly our number of matched BBB and tumors is limited, the data from these specimens suggests that, later in oncogenesis, mutations in *ATR* pathway members, i.e., *ATR* and *ETAA1*, are being selected for as they observed in both the benign biopsy and its matched tumor. We note that *ATR* haploinsufficieny in a mismatch repair deficient background has been shown to result in dramatic increases in fragile site instability, amplifications and rearrangements, and in decreased tumor latency^65^.

In summary, we have taken an initial step towards what will be a series of investigations of somatic DNA changes in the unaffected breast, which will help define alterations that put women at substantially elevated BC risk. Such studies will also provide the possibility of estimating the time frame of that risk, so that women are able to make practical decisions regarding the interventions that they choose to adopt. We have shown that such work is feasible, with sequencing quality that meets current standards in the field, that somatic sequencing data can be inferred and interpreted even in the absence of matched germline data, and those intriguing findings emerge that are cancer relevant.

## Methods

### Sample collection

At the Northwestern Feinberg School of Medicine, we designed a case-control study of BBB samples (BBCAR Study)^66^. Subjects were identified through searches of the Enterprise Data Warehouse of Northwestern Medicine (NM), and at the Lynn Sage Breast Center of NM, under IRB-approved protocol NU 09B2. The major eligibility criterion required a history of benign breast biopsy performed at NM, at least 1 year prior to cancer diagnosis for cases. Eligible subjects provided written informed consent for the use of their BBB blocks after the nature and possible consequences of the study were explained, and completed a survey detailing breast history and breast cancer risk factors. We have retrieved the BBB paraffin blocks of subjects who subsequently developed breast cancer (cases) and from age-matched controls, who have not developed breast cancer to date. The participants are contacted periodically to confirm that controls have not transitioned to cases. A subset of 135 cases, matched to 69 controls were selected for WES (“Supplementary Materials and Methods”). All subjects included in this analysis were of European descent. Case and control samples are matched by age and histologic class (non-proliferative benign change, or proliferation without atypia). DNA was isolated from the LCM epithelium and sequenced using the Illumina HiSeq4000. WES was conducted with a sequencing depth of 80–100× and 80–90 million sequencing reads were generated for each sample (Supplementary Materials and Methods).

### Parallel alignment of whole-exome analysis

We adapted widely used open source software for genome alignment and variant calling. Read alignment and variant calling were performed according to the Broad Institute’s Genome Analysis Toolkit (GATK) best practices pipeline^67^. Reads were aligned to the human reference genome (hg19) using Burrows-Wheeler alignment^68^ and Picard 2.6 was subsequently used to sort reads and mark duplicates (Fig. 1c). To reduce systematic errors, sorted BAM files were separately generated based on the sequence lane that the reads were generated. By doing so, various technical artifacts that are associated with lane-specific artifacts can be removed during duplicate marking and base recalibration steps. Base recalibration was done using the GATK 3.6 using dbSNP build 138 as a training set. Mutations were called and filtered using MuTect2 in the GATK package. To capture recurrent technical artifacts, we generated a Panel of Normals (PON) for Mutect2 analysis using the sequenced 26 germline DNA. The PON is created by running the variant caller Mutect2 individually on the normal samples and combining the resulting variant calls with the criteria of excluding any sites that are not present in at least 2 normals, which is the default cutoff^69^. For the samples without matched normal DNA available, we run Mutect2 using the so called “tumor only” model with PON filtering to call mutations. To obtain a set of mutations with the highest sensitivity, VarScan2^35^ and VarDict^70^ were also applied for mutation calling. To further ensure a high precision call rate, we filtered all mutations with read depth <20. After filtering, mutations were then annotated using SNPEFF^71^, VEP^72^, and ANNOVAR^25^.

### Somatic mutation identification

Our initial objective was to develop and test a predictive model for somatic mutation identification. A significant challenge for this study, and for others seeking to identify somatic mutations in archived tissue samples is the lack of matched germline DNA. Therefore, to prepare for ground truth, previously consented donors were re-contacted (with IRB approval) and saliva specimens were requested for normal DNA sequencing. Matched germline DNA was obtained for 26 of the 204 BBB specimens which had been selected for WES. For these 26 paired samples, a set of somatic mutations were generated by using Mutect2 tumor-normal pair mode with PON filtering. Independently, for these BBB samples, a set of mutations were generated using Mutect2 tumor-only mode with PON filtering. This is the mode to be used for the rest of BBBs without matched normal DNA. However, mutations generated in this mode contain germlines variants. To rule these germline variants, we overlapped this set mutations with their BBB’s germline variants, which were generated using GATK Haplotype callers. The overlapped variants were then labeled as germline variants, together with the somatic mutations were used for model evaluation. We systematically evaluated multiple machine learning models and adopted Multi-Layer Perceptron (MLP) for somatic mutation classification. Features in the prediction model included intrinsic sequencing features, such as mutation allele frequency, depth of reference reads, number of appearances in the cohort as well as published collated data providing the frequency of the variant in the population and predictions of the impact of amino acid changes on the structure and function of the encoded protein. The model obtained an accuracy of 95% for somatic mutation in the test set (“Supplementary Materials and Methods”). Orthogonal SNP array genotyping was performed to compare and validate the performance of mutation calling and mutation classification. Technical validation was performed for 17 of the 26 specimens for which matched germline data was available, and 3 of the specimens without matched germline, using the Infinium Exome-24 v1.1 beadchip (“Supplementary Materials and Methods”). The case group is defined as benign biopsies that developed breast cancer at least one year later after the biopsy. In the case group, we have retrieved 10 cancer blocks that matched to the cases (Fig. 1b). The same preprocessing procedures were performed as benign biopsies, including LCM dissection, DNA extraction, library construction, sequencing, alignment, mutation calling, and filtering.

### Somatic copy number variation and mutational signature

Using both aligned reads and identified mutations, we studied the genetic aberrations that distinguish cases from controls, including mutations and CNVs. We identified the somatic mutations or CNVs that were common to both the cases’ benign biopsy tissue as well as paired malignant lesions for the ten cases in which we had both tissues available. *P*-values were derived with the use of Chi-square test or logistic regression. We also studied the mutations to enable the discovery of mutational signatures. Lastly, we evaluated machine learning models and features for breast cancer risk prediction for the cohort. Benjamini-Hochberg method was applied to convert the two-sided *P*-values to False Discover Rate (FDR) for multi-comparison correction.

A Mutational Signature study was performed to reveal underlying mutational processes for cancer development. The identified somatic mutations were presented as a 96-element vector, which captures the immediate 5′ and 3′ neighbors of the mutated nucleotides. The summary of these mutation characteristics forms a mutational profile for each tissue sample. Putting multiple samples’ profiles together form a matrix with the number of samples as rows (204) and the mutation characteristics as columns (96). Nonnegative matrix factorization (NMF) was applied to enable the discovery of intrinsic patterns in this matrix. The first value where the Residual Sum of Squares (RSS) curve presents an inflection point was used to determine the number of signatures. In total, three signatures were discovered among the cases and controls, or combined. The outputs of NMF consist of an H matrix and a W matrix. The matrix H (dimension of 3 × 96) was used to infer mutational processes. The numbers in matrix W (dimension of 204 × 3) correspond to each samples’ signature exposure levels. This matrix was interpreted as each tissue sample’s accumulated exposure effect to the mutational burden. We further evaluated the association between the signature exposure level and cancer development with logistic regressions, adjusting for age and histology class.

### Cancer risk prediction at BBB

To predict cancer development using the mutations identified in BBB, we fit logistic regression with L1 penalty using the case/control status as output variable. Multiple input features have been tested, namely, clinical risk factors, somatic mutations, mutation burden by gene/cytoband/protein domain. The mutation burden is inferred by aggregating all somatic mutations annotated as same gene/cytoband/protein domain to a continuous number, representing the mutation burden of the corresponding unit. In a cross-comparison evaluation, we achieved the best results using protein domains as aggregation unit. In total, 1966 annotated protein domains were utilized as input features for case/control prediction (Supplementary data 5)^22^. To evaluate the model and features, we performed a bootstrapping by randomly splitting the BBB samples at a 7:3 ratio for training and testing. We also evaluated the models by including clinical risk factors, including age at the time of BBB, age at menarche, age at first live birth, family history of BC in a first-degree relative, histologic variable (proliferative vs non-proliferative).

### Reporting summary

Further information on research design is available in the Nature Research Reporting Summary linked to this article.

## Supplementary information

Supplementary Materials and Methods

Reporting Summary

## Data Availability

The datasets generated and analysed during the current study are publicly available in the figshare repository: 10.6084/m9.figshare.12191793^22^. Whole-exome sequencing data, generated during the current study, are publicly available in NCBI Sequence Read Archive (SRA) here: https://identifiers.org/insdc.sra:SRP219328^23^. TCGA data supporting Fig. 2, were downloaded from the Genomic Data Commons (GDC) data portal, though a dbGaP application. The link to the relevant dbGaP study is https://identifiers.org/dbgap:phs000178.v1.p1.

## Code availability

All codes necessary to process the sequencing data and to re-generate the results are publicly available at https://github.com/zexian/BBCAR_codes^22^.

## References

[1] Siegel RL, Miller KD, Jemal A (2019). Cancer statistics, 2019. CA: a cancer J. clinicians.

[2] Flanagan, M. R., et al. Chemoprevention Uptake for Breast Cancer Risk Reduction Varies by Risk Factor. *Ann. Surg. Oncol.*10.1245/s10434-019-07236-8 (2019). 10.1245/s10434-019-07236-8 PMC6545244 30815800

[3] Gail MH (1989). Projecting individualized probabilities of developing breast cancer for white females who are being examined annually. J. Natl Cancer Inst..

[4] Chlebowski RT (2007). Predicting risk of breast cancer in postmenopausal women by hormone receptor status. J. Natl Cancer Inst..

[5] Pankratz VS (2008). Assessment of the accuracy of the Gail model in women with atypical hyperplasia. J. Clin. Oncol..

[6] Boughey JC (2010). Evaluation of the Tyrer-Cuzick (International Breast Cancer Intervention Study) model for breast cancer risk prediction in women with atypical hyperplasia. J. Clin. Oncol..

[7] Dupont WD, Page DL (1985). Risk factors for breast cancer in women with proliferative breast disease. N. Engl. J. Med..

[8] Dupont WD (1993). Breast cancer risk associated with proliferative breast disease and atypical hyperplasia. Cancer.

[9] Visscher DW (2016). Clinicopathologic features of breast cancers that develop in women with previous benign breast disease. Cancer.

[10] Silverstein MJ (2009). Special report: consensus conference III. Image-detected breast cancer: state-of-the-art diagnosis and treatment. J. Am. Coll. Surg..

[11] Ju YS (2017). Somatic mutations reveal asymmetric cellular dynamics in the early human embryo. Nature.

[12] Alexandrov LB (2015). Clock-like mutational processes in human somatic cells. Nat. Genet.

[13] Knudson AG (2001). Two genetic hits (more or less) to cancer. Nat. Rev. Cancer.

[14] Tamborero D (2013). Comprehensive identification of mutational cancer driver genes across 12 tumor types. Sci. Rep..

[15] Danforth DN (2016). Genomic changes in normal breast tissue in women at normal risk or at high risk for breast cancer. Breast Cancer (Auckl.).

[16] Sakr RA (2016). Targeted capture massively parallel sequencing analysis of LCIS and invasive lobular cancer: repertoire of somatic genetic alterations and clonal relationships. Mol. Oncol..

[17] Martincorena I (2015). High burden and pervasive positive selection of somatic mutations in normal human skin. Science.

[18] Stratton MR, Campbell PJ, Futreal PA (2009). The cancer genome. Nature.

[19] Alexandrov LB (2013). Signatures of mutational processes in human cancer. Nature.

[20] Temko D, Tomlinson IPM, Severini S, Schuster-Bockler B, Graham TA (2018). The effects of mutational processes and selection on driver mutations across cancer types. Nat. Commun..

[21] Hartmann LC (2005). Benign breast disease and the risk of breast cancer. N. Engl. J. Med..

[22] Zeng, Z., et al. Datasets and metadata supporting the published article: somatic genetic aberrations in benign breast disease and the risk of subsequent breast cancer. *figshare.*10.6084/m6089.figshare.12191793 (2020). 10.1038/s41523-020-0165-z PMC7293275 32566745

[23] NCBI Sequence Read Archive https://identifiers.org/insdc.sra:SRP219328 (2020).

[24] Flanagan SE, Patch A-M, Ellard S (2010). Using SIFT and PolyPhen to predict loss-of-function and gain-of-function mutations. Genet. Test. Mol. Biomark..

[25] Wang K, Li M, Hakonarson H (2010). ANNOVAR: functional annotation of genetic variants from high-throughput sequencing data. Nucleic Acids Res..

[26] Kalatskaya I (2017). ISOWN: accurate somatic mutation identification in the absence of normal tissue controls. Genome Med..

[27] Bailey MH (2018). Comprehensive characterization of cancer driver genes and mutations. Cell.

[28] Sharma Y (2019). A pan-cancer analysis of synonymous mutations. Nat. Commun..

[29] Pearlman R (2017). Prevalence and spectrum of germline cancer susceptibility gene mutations among patients with early-onset colorectal cancer. JAMA Oncol..

[30] Hanisch FG (2001). O-glycosylation of the mucin type. Biol. Chem..

[31] Rohan TE (2018). Somatic mutations in benign breast disease tissue and risk of subsequent invasive breast cancer. Br. J. Cancer.

[32] Nik-Zainal S (2016). Landscape of somatic mutations in 560 breast cancer whole-genome sequences. Nature.

[33] Petljak M (2019). Characterizing mutational signatures in human cancer cell lines reveals episodic APOBEC mutagenesis. Cell.

[34] Roberts SA (2013). An APOBEC cytidine deaminase mutagenesis pattern is widespread in human cancers. Nat. Genet.

[35] Koboldt, D. C., et al. VarScan 2: somatic mutation and copy number alteration discovery in cancer by exome sequencing. *Genome Res.* 22, 568–576 (2012). 10.1101/gr.129684.111 PMC3290792 22300766

[36] Mermel CH (2011). GISTIC2. 0 facilitates sensitive and confident localization of the targets of focal somatic copy-number alteration in human cancers. Genome Biol..

[37] Bass TE (2016). ETAA1 acts at stalled replication forks to maintain genome integrity. Nat. Cell Biol..

[38] Curtis C (2012). The genomic and transcriptomic architecture of 2,000 breast tumours reveals novel subgroups. Nature.

[39] Ciriello G (2013). Emerging landscape of oncogenic signatures across human cancers. Nat. Genet..

[40] Davies H (2017). Whole-genome sequencing reveals breast cancers with mismatch repair deficiency. Cancer Res.

[41] Soysal SD (2019). Genetic alterations in benign breast biopsies of subsequent breast cancer patients. Front Med. (Lausanne).

[42] Pankratz VS (2015). Model for individualized prediction of breast cancer risk after a benign breast biopsy. J. Clin. Oncol..

[43] Spencer DH (2013). Comparison of clinical targeted next-generation sequence data from formalin-fixed and fresh-frozen tissue specimens. J. Mol. Diagn..

[44] Bhagwate AV (2019). Bioinformatics and DNA-extraction strategies to reliably detect genetic variants from FFPE breast tissue samples. BMC Genomics.

[45] Robbe P (2018). Clinical whole-genome sequencing from routine formalin-fixed, paraffin-embedded specimens: pilot study for the 100,000 Genomes Project. Genet Med..

[46] Wang Y (2014). Clonal evolution in breast cancer revealed by single nucleus genome sequencing. Nature.

[47] Soysal SD (2015). Status of estrogen receptor 1 (ESR1) gene in mastopathy predicts subsequent development of breast cancer. Breast Cancer Res Treat..

[48] Xia Y, Fan C, Hoadley KA, Parker JS, Perou CM (2019). Genetic determinants of the molecular portraits of epithelial cancers. Nat. Commun..

[49] Bartkova J (2006). Oncogene-induced senescence is part of the tumorigenesis barrier imposed by DNA damage checkpoints. Nature.

[50] Minnick DT, Pavlov YI, Kunkel TA (1994). The fidelity of the human leading and lagging strand DNA replication apparatus with 8-oxodeoxyguanosine triphosphate. Nucleic Acids Res.

[51] Colussi C (2002). The mammalian mismatch repair pathway removes DNA 8-oxodGMP incorporated from the oxidized dNTP pool. Curr. Biol..

[52] Pursell ZF, McDonald JT, Mathews CK, Kunkel TA (2008). Trace amounts of 8-oxo-dGTP in mitochondrial dNTP pools reduce DNA polymerase gamma replication fidelity. Nucleic Acids Res.

[53] Freudenthal BD (2015). Uncovering the polymerase-induced cytotoxicity of an oxidized nucleotide. Nature.

[54] Garrido-Castro AC, Lin NU, Polyak K (2019). Insights into molecular classifications of triple-negative breast cancer: improving patient selection for treatment. Cancer Disco..

[55] Lim E (2009). Aberrant luminal progenitors as the candidate target population for basal tumor development in BRCA1 mutation carriers. Nat. Med.

[56] Molyneux G (2010). BRCA1 basal-like breast cancers originate from luminal epithelial progenitors and not from basal stem cells. Cell Stem Cell.

[57] Kannan N (2014). Glutathione-dependent and -independent oxidative stress-control mechanisms distinguish normal human mammary epithelial cell subsets. Proc. Natl Acad. Sci. USA.

[58] Tomkova M, McClellan M, Kriaucionis S, Schuster-Bockler B (2018). DNA Replication and associated repair pathways are involved in the mutagenesis of methylated cytosine. DNA Repair (Amst.).

[59] Chen PC (2008). Novel roles for MLH3 deficiency and TLE6-like amplification in DNA mismatch repair-deficient gastrointestinal tumorigenesis and progression. PLoS Genet.

[60] Morris LG (2013). Recurrent somatic mutation of FAT1 in multiple human cancers leads to aberrant Wnt activation. Nat. Genet.

[61] Martin D (2018). Assembly and activation of the Hippo signalome by FAT1 tumor suppressor. Nat. Commun..

[62] Li Z (2018). Loss of the FAT1 tumor suppressor promotes resistance to CDK4/6 inhibitors via the hippo pathway. Cancer Cell.

[63] Cimprich KA, Cortez D (2008). ATR: an essential regulator of genome integrity. Nat. Rev. Mol. Cell Biol..

[64] Casper AM, Nghiem P, Arlt MF, Glover TW (2002). ATR regulates fragile site stability. Cell.

[65] Fang Y (2004). ATR functions as a gene dosage-dependent tumor suppressor on a mismatch repair-deficient background. EMBO J..

[66] Shidfar A (2017). Expression of miR-18a and miR-210 in normal breast tissue as candidate biomarkers of breast cancer risk. Cancer Prev. Res. (Phila.).

[67] McKenna A (2010). The Genome Analysis Toolkit: a MapReduce framework for analyzing next-generation DNA sequencing data. Genome Res.

[68] Li H, Durbin R (2010). Fast and accurate long-read alignment with Burrows-Wheeler transform. Bioinformatics.

[69] Cibulskis K (2013). Sensitive detection of somatic point mutations in impure and heterogeneous cancer samples. Nat. Biotechnol..

[70] Lai Z (2016). VarDict: a novel and versatile variant caller for next-generation sequencing in cancer research. Nucleic acids Res..

[71] Cingolani P (2012). A program for annotating and predicting the effects of single nucleotide polymorphisms, SnpEff: SNPs in the genome of Drosophila melanogaster strain w1118; iso-2; iso-3. Fly (Austin).

[72] McLaren W (2016). The ensembl variant effect predictor. Genome Biol..

