# Supplementary material for: Somatic genetic aberrations in benign breast disease and the risk of subsequent breast cancer
Source: NPJ Breast Cancer. 2020 Jun 12;6:24. doi: 10.1038/s41523-020-0165-z (PMC7293275; doi:10.1038/s41523-020-0165-z)
Supplement: Supplementary file 1 — Supplementary Materials and Methods [file 41523_2020_165_MOESM1_ESM.pdf]

Somatic Genetic Aberrations in Benign Breast Disease and the Risk of Subsequent Breast Cancer

Zexian Zeng[1,2], Andy Vo[3], Xiaoyu Li[4], Ali Shidfar[5], Paulette Saldana[5], Luis Blanco[6], Xiaoling Xuei[7], Yuan Luo[1*], Seema A. Khan[5*], Susan E. Clare[5*]

Supplementary Materials and Methods

Supplementary Table 1: **List of features used in the predictive model for somatic mutation identification.** 'Allele frequency' is the mutation allele frequency, 'Ref depth' is the read depth of the reference allele, 'Fre cohort' is the number of appearances of this mutation in the cohort. SNP common is a binary variable indicating that the mutation appears in the database of dbSNP after removing those flagged SNPs (SNPs < 1% minor allele frequency (MAF) (or unknown), mapping only once to reference assembly, flagged in dbSNP as "clinically associated"). 'COSMIC' is a binary variable indicating whether the mutation appears in the COSMIC version 80. The other features were derived from functional annotations from functional annotation tools.

Allele frequency	Polyphen2 HVAR pred	MetaLR score
Ref depth	LRT score	MetaLR pred
Freq cohort	LRT pred	VEST3 score
SNP common	Mutation Taster score	CADD raw
ExAC	Mutation Taster pred	CADD phred
COSMIC	Mutation Assessor score	GERP score
SIFT score	Mutation Assessor pred	phyloP20 way mammalian
SIFT pred	FATHMM score	phyloP100 way vertebrate
Polyphen2 HDIV score	FATHMM pred	SiPhy 29 way logOdds
Polyphen2 HDIV pred	MetaSVM score	
Polyphen2 HVAR score	MetaSVM pred	

Supplementary Table 2: **The number of mutations in the train set and test set and the prediction performance achieved in the multiple layer perceptron (MLP).** The germline variants were treated as predictive negative and somatic mutations were treated as predictive positive. Using tuned multiple layer perceptron model to predict mutations in the holdout test set, the AUC score, precision, recall, and F-Measure are reported.

		**Train Set**	**Test Set**
	Germline	10464	3315
	Somatic	2817	1115
**MLP**			

**AUC**	Precision	Recall	F1-score
**0.98**	0.95	0.98	0.96

Supplementary Table 3: **The number of missense mutations within MUC17 that resulted in the gain or loss of either serine or threonine residues**. While 8.7% of missense mutations in MUC17 would be predicted to result in the loss of serine, 16.8% in the loss of threonine, 14.2% in the gain of serine and 17.8% in the gain of threonine, there was no significant difference in between cases and controls. Pearson's Chi-squared tests were performed for categorical variables.

	**Serine lose**	**Serine gain**	**Threonine lose**	**Threonine gain**
**Case**	19 (10.9%)	23 (13.2%)	31 (17.8%)	25 (14.4%)
**Control**	8 (5.9%)	21 (15.5%)	21 (15.5%)	30 (22.2%)
**Sum**	27 (8.7%)	44 (14.2%)	52 (16.8%)	55 (17.8%)
**P-value**	0.12	0.56	0.60	0.07

Supplementary Table 4: **The ten most frequently mutated genes shared between the BBB of cases and their tumors.** TSG=**tumor suppressor** *OG=**oncogene**

	**Description**	**N**	**Consensus**
FAT1	FAT Atypical Cadherin 1	8	TSG
EPG5	Ectopic P-granules autophagy protein 5	7	
BZRAP1	TSPO associated protein 1	6	
CTNNA2	Catenin Alpha2	6	OG, TSG
MYH11	Myosin Heavy chain 11	6	
ABCA13	ATP binding cassette subfamily A	5	
ATR	ATR Serine/Threonine Kinase	5	TSG
DNAH14	Dynein axonemal heavy chain 14	5	
ETAA1	ETAA1 activator of ATR kinase	5	
FBXL18	F-box and leucine rich repeat protein 18	5	

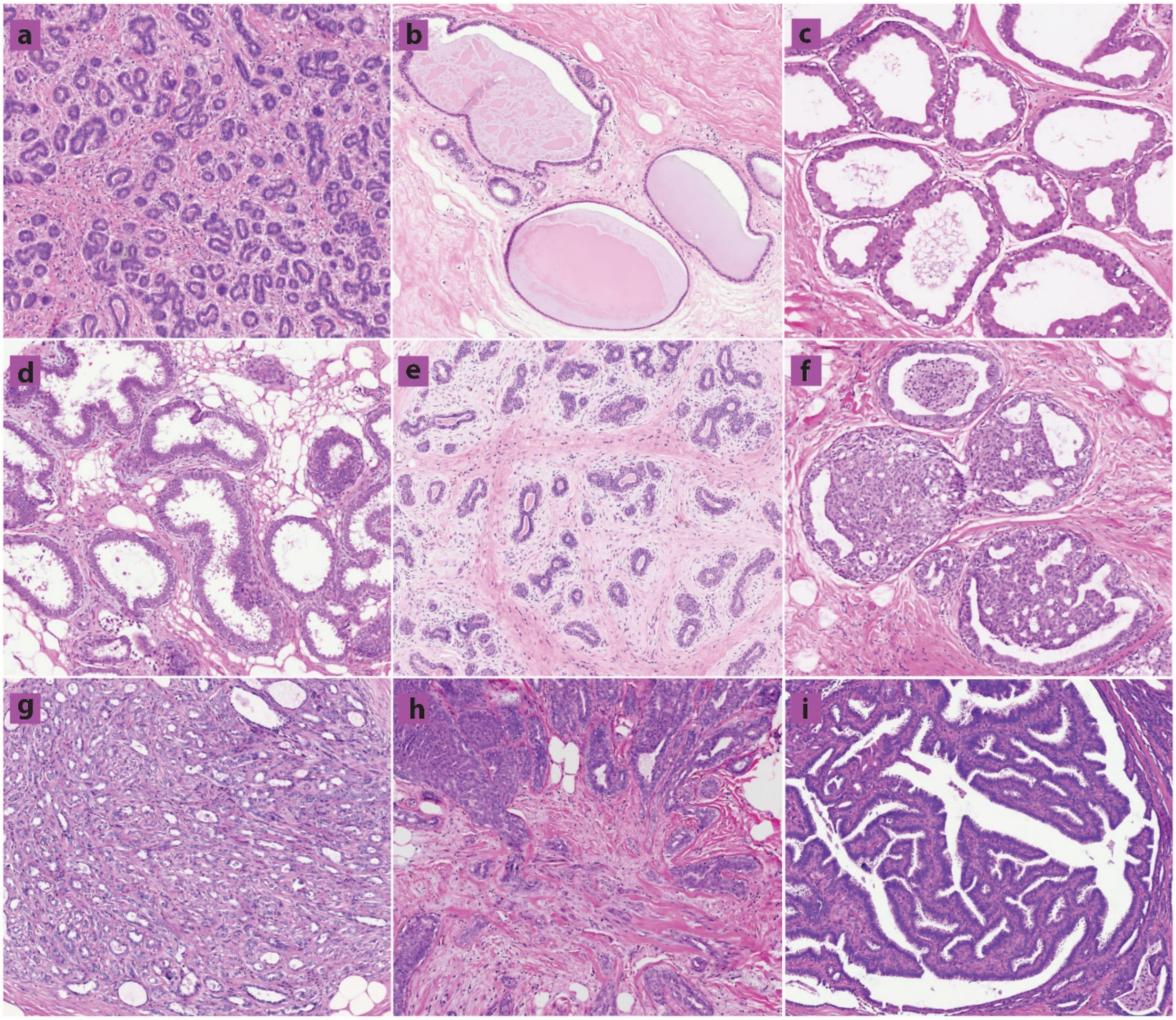

Supplementary Figure 1: **Examples of benign non-atypical breast lesions**. Nonproliferative lesions included: (a) adenosis – lobule with increased number of acini with open lumens, (b) cysts – dilated ducts lined by flattened epithelial cells, (c) apocrine metaplasia – cystically dilated ducts lined by epithelial cells with abundant granular eosinophilic cytoplasm and round nuclei, (d) columnar cell change – dilated glands lined by tall cells with ovoid nuclei perpendicular to the basement membrane, and (e) fibroadenoma without epithelial hyperplasia – biphasic proliferation of benign epithelium and surrounding intralobular stroma. Proliferative lesions included: (f) florid ductal hyperplasia without atypia – distended ducts lined by increased numbers of a mixed population of luminal and myoepithelial cells with irregular slit like fenestrations, (g) sclerosing adenosis – enlarged terminal duct lobular unit with increased number of acini that are compressed and distorted by dense stroma, (h) radial scar – central nidus of hyalinized stroma with entrapped glands surrounded by long radiating projections containing epithelium with varying degrees of cyst formation and hyperplasia, and (i) papilloma – dilated duct with multiple branching fibrovascular cores lined by myoepithelial and epithelial cells.

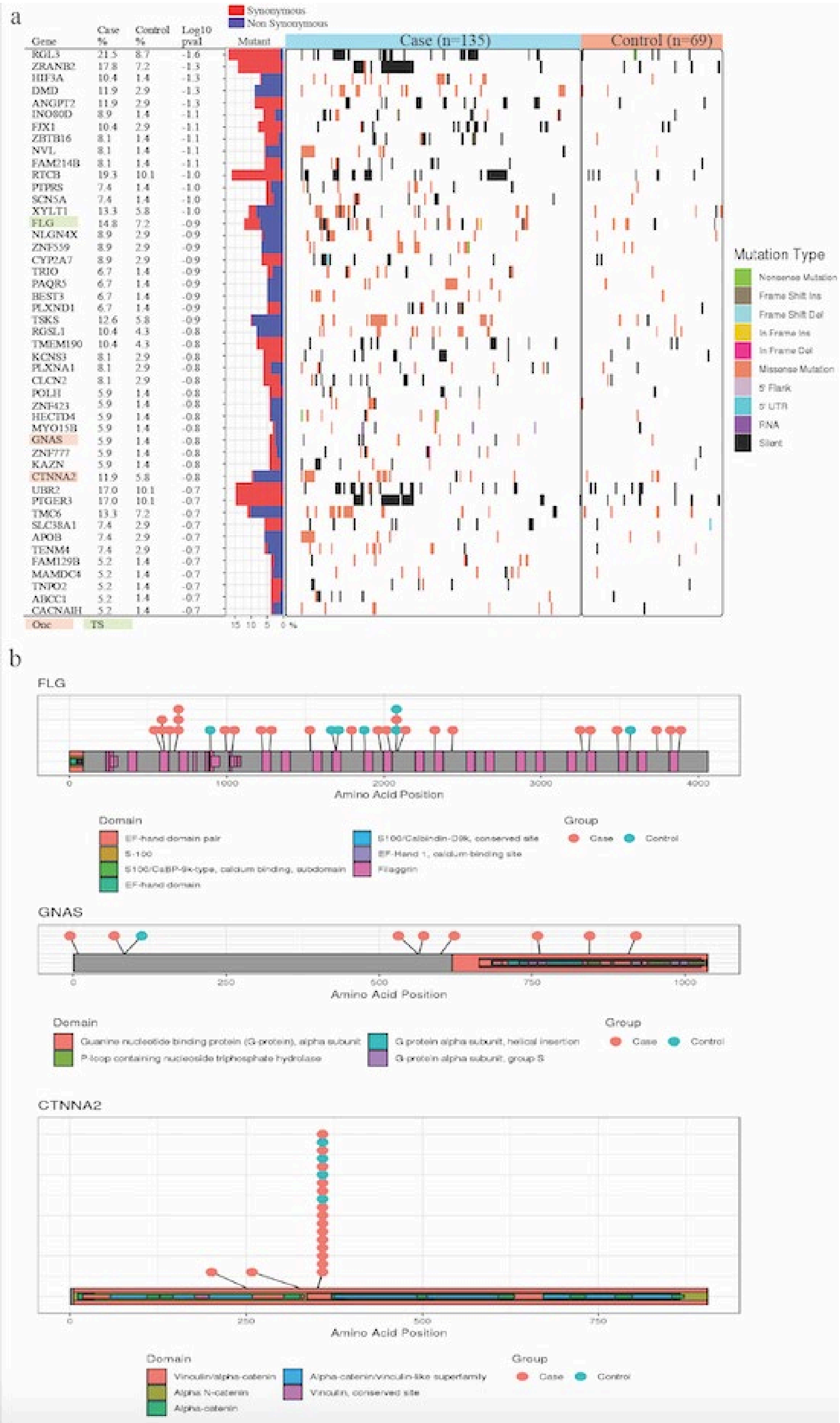
a
Synonymous
Non Synonymous
Gene
Case %
Control %
Log10 pval
Mutant
Case (n=135)
Control (n=69)
RGL3 21.5 8.7 -1.6
ZRANB2 17.8 7.2 -1.3
HIF3A 10.4 1.4 -1.3
DMD 11.9 2.9 -1.3
ANGPT2 11.9 2.9 -1.3
INO80D 8.9 1.4 -1.1
FJX1 10.4 2.9 -1.1
ZBTB16 8.1 1.4 -1.1
NVL 8.1 1.4 -1.1
FAM214B 8.1 1.4 -1.1
RTCB 19.3 10.1 -1.0
PTPRS 7.4 1.4 -1.0
SCN5A 7.4 1.4 -1.0
XYLT1 13.3 5.8 -1.0
FLG 14.8 7.2 -0.9
NLGN4X 8.9 2.9 -0.9
ZNF559 8.9 2.9 -0.9
CYP2A7 8.9 2.9 -0.9
TRIO 6.7 1.4 -0.9
PAQR5 6.7 1.4 -0.9
BEST3 6.7 1.4 -0.9
PLXND1 6.7 1.4 -0.9
TSKS 12.6 5.8 -0.9
RGSL1 10.4 4.3 -0.8
TMEM190 10.4 4.3 -0.8
KCNS3 8.1 2.9 -0.8
PLXNA1 8.1 2.9 -0.8
CLCN2 8.1 2.9 -0.8
POLH 5.9 1.4 -0.8
ZNF423 5.9 1.4 -0.8
HECTD4 5.9 1.4 -0.8
MYO15B 5.9 1.4 -0.8
GNAS 5.9 1.4 -0.8
ZNF777 5.9 1.4 -0.8
KAZN 5.9 1.4 -0.8
CTNNA2 11.9 5.8 -0.8
UBR2 17.0 10.1 -0.7
PTGER3 17.0 10.1 -0.7
TMC6 13.3 7.2 -0.7
SLC38A1 7.4 2.9 -0.7
APOB 7.4 2.9 -0.7
TENM4 7.4 2.9 -0.7
FAM129B 5.2 1.4 -0.7
MAMDC4 5.2 1.4 -0.7
TNPO2 5.2 1.4 -0.7
ABCC1 5.2 1.4 -0.7
CACNA1H 5.2 1.4 -0.7
Onc
TS
15 10 5 0 %
Mutation Type
Nonsense Mutation
Frame Shift Ins
Frame Shift Del
In Frame Ins
In Frame Del
Missense Mutation
5' Flank
5' UTR
RNA
Silent
b
FLG
0 1000 2000 3000 4000
Amino Acid Position
Domain
EF-hand domain pair
S-100
S100/CaBP-9k-type, calcium binding, subdomain
EF-hand domain
S100/Calbindin-D9k, conserved site
EF-Hand 1, calcium-binding site
Filaggrin
Group
Case
Control
GNAS
0 250 500 750 1000
Amino Acid Position
Domain
Guanine nucleotide binding protein (G-protein), alpha subunit
P-loop containing nucleoside triphosphate hydrolase
G-protein alpha subunit, helical insertion
G-protein alpha subunit, group S
Group
Case
Control
CTNNA2
0 250 500 750
Amino Acid Position
Domain
Vinculin/alpha-catenin
Alpha N-catenin
Alpha-catenin
Alpha-catenin/vinculin-like superfamily
Vinculin, conserved site
Group
Case
Control

Supplementary Figure 2: **Genetic aberrations that distinguish case and control and mutational positions. a.** For each gene, the percentage of mutated individuals in the case and control were shown. Onc are known oncogenes; TS are known tumor suppressor genes. The p-values were derived using the case/control as output and the mutated individual as inputs in logistic regression. The middle panel shows the synonymous versus nonsynonymous rate. In the right panel, each column is an individual, and the color represents the mutation class. **b.** Position of mutational alterations in the protein structure of FLG, GNAS, and CTNNA2.

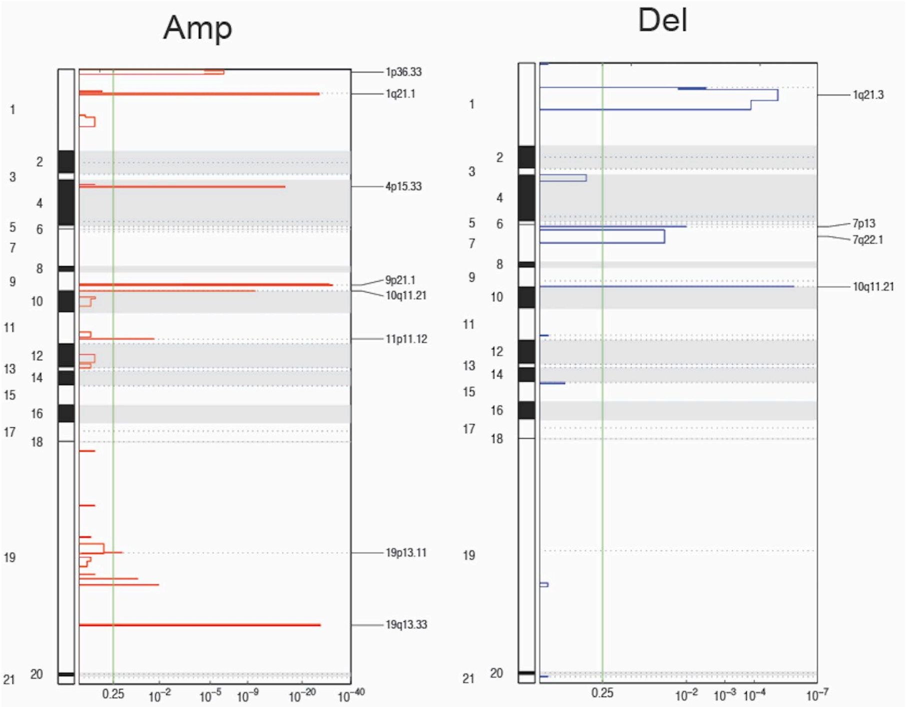

Supplementary Figure 3: **Genome wide amplifications and deletions among the 20 cases with matched normal DNA. Left: amplification. Right, deletion**. The results were generated by VarScan2 and GISTIC2.

Pathology/Histology

Benign breast diseases were classified based on previously established categories [1-3] according to subsequent risk of developing breast cancer as follows: (1) nonproliferative, (2) proliferative without atypia, or (3) proliferative with atypia. Nonproliferative lesions included cases with a diagnosis of adenosis, cysts, apocrine metaplasia, fibroadenoma without epithelial hyperplasia, columnar cell change, and mild ductal hyperplasia of the usual type (Supplementary Figure 1, a-e). Proliferative lesions without atypia included cases with a diagnosis of moderate to florid ductal hyperplasia of the usual type, papilloma, sclerosing adenosis, and complex sclerosing

lesions including radial scar (Supplementary Figure 1, f-i). Proliferative lesions with atypia included cases with a diagnosis of atypical ductal hyperplasia (ADH) and atypical lobular hyperplasia (ALH).

Library construction and sequencing

Ten 10-micron sections per sample were cut from formalin-fixed, paraffin-embedded (FFPE) tissue blocks, and the matched areas of interest isolated by laser capture microdissection (LCM). In detail, we took slides to Center for Advanced Microscopy (CAM) and used Zeiss Palm microscope to micro-dissect and collect areas of interest in a 500μl adhesive cap (AdhesiceCap 500 opaque -Zeiss order number 415190-9201-000). Total genomic DNA was extracted from the LCM samples, using Qiagen AllPrep DNA/RNA FFPE it (Cat. No. 80234). DNA concentration was measured by Nanodrop. Samples used for this study yielded >300ng of DNA. The concentration and quality of gDNA samples were first assessed using Agilent 4200 TapeStation. Then 100-200 nanograms of DNA per sample were used to prepare single-indexed cDNA library using SureSelectXT Human All Exon V6 (58Mb) (Agilent). The resulting libraries were assessed for its quantity and size distribution using Qubit and Agilent 2100 Bioanalyzer. Two hundred pico molar per liter pooled libraries were utilized per flow cell for clustering amplification on cBot using HiSeq 3000/4000 PE Cluster Kit and sequenced with 2×75bp paired-end configuration on HiSeq4000 (Illumina) using HiSeq 3000/4000 PE SBS Kit. A Phred quality score (Q score) was used to measure the quality of sequencing. More than 90% of the sequencing reads reached Q30 (99.9% base call accuracy). With the goal of sequencing at 100X, the final average sequencing depth was 69X, and there were 80-90 million sequencing reads per sample. To note, after the first

round of sequencing, 27 samples had the coverage under 50X, and were re-sequenced for deeper coverage under the same protocol.

Microarray genotyping

To evaluate the performance of called somatic mutations, a subset of samples was separately LCM dissected, and the extracted DNA were genotyped using Infinium Exome-24 Kit, which covers 240,000 markers in a catalog of exome variants. Of note, the quality control sample had a call rate of 99.34%. During the QC period, no samples failed restoration. For quality control, three samples were repeatedly genotyped twice, and R-square rates were calculated for the overlap. The reported R-square rates are 98.86%, 99.29%, and 99.39%. The high overlap rates indicate a high stability of calling variants from our DNA. Genotyped variants from the 17 samples were mapped to genomic assemblies (hg19). To note, only the calls with GCScore larger than 0.15 were retained. The coordinates that appear both in genotype array and somatic mutations called by MuTect2, VarScan2, or VarDict were retrieved. The allele frequencies derived from both technologies were compared. The overlap number between array and MuTec2, VarScan2, VarDict are 384, 963, and 124,511 respectively.

Classification model for somatic mutations

Our initial objective was to develop and test a predictive model for somatic mutation identification. MuTect2 is known as one of the most reliable and sensitive cancer somatic mutation callers.[4] We have learned that mutations identified by MuTect2 have higher accuracy than the other callers. In this study, MuTect2 was used to call somatic mutations from the 26 benign biopsies and matched normal germline DNA. To reduce the false positive call rates, the following mutations were labeled

as germline variants: those that appear in dbSNP with ANNOVAR[5] index files (after removing those SNPs < 1% minor allele frequency (or unknown), or mapping only once to reference assembly, or flagged in dbSnp as "clinically associated") and not in COSMIC database (version 80). The called mutations from these 26 matched samples were used as the gold standard for the predictive model training and testing. The 28177 called mutations were randomly split to cross-validation set and holdout test set based on a 7:3 ratio.

Tools predicting somatic mutations for germline DNA free samples have been developed. However, the developed tools attempted to predict somatic mutations in tumor-only samples. Tools that have been developed and validated using the mutations derived from tumor samples cannot be applied to benign biopsies directly, mostly due to the different feature landscapes in mutations derived from tumors and benign biopsies. For example, allele frequency derived in tumor samples are expected to be higher than allele frequencies in benign biopsies[6] In order to predict somatic mutations in the benign-only biopsies without matched normal DNA, in this study, we attempted to develop and evaluate a new predictive model to predict somatic mutations in benign biopsies.

In total, 31 features (Supplementary Table 1) were retrieved or developed to create the predictive model for somatic mutation identification. Multiple tools have been developed for potential pathogenicity prediction. These tools consider either the protein structure, population frequency, or evolutionary factors.[7] Various functional annotation or toxicity scores were derived from ANNOVAR,[5] COSMIC (https://cancer.sanger.ac.uk/cosmic), dbSNP/common (https://www.ncbi.nlm.nih.gov), along with intrinsic sequencing features, such as mutation allele frequency, depth of reference reads, mutation frequency in the cohort. Not all mutations were annotated in each of the database. However, missing data that appear in more than one feature

could challenge some of the classifiers (e.g. logistic regression). Considering that the features are a mix of continuous number, binary feature, and categorical variables, Multivariate Imputation by Chained Equations (MICE)[8] was used to impute the missing values. In detail, 20 sets of data were imputed with the iteration equal to 20. Utilizing the derived features, we evaluated multiple linear and nonlinear machine learning models for somatic mutation classification. Grid search was applied to tune each model's parameters using five-fold cross-validation on the training set. To reduce the risk of having false positives, precision was used as selection criteria for parameter tuning and model selection. Once the model was tuned, it was applied on the held-out test set for precision, recall, F-measure, and AUC score reporting. The model with the highest precision was selected as our somatic mutation predictive model. To maximize the prediction power, we evaluated multiple machine learning methods, including penalized logistic regression (LR), linear SVM, random forest classifier (RFC), gradient boosted tree (GBT), k-nearest neighbor algorithm (K-NN), SVM with rbf kernel, and multi-layer perceptron (MLP). With the parameters tuned, the models were evaluated within the holdout test. The machining learning models achieved different performances in the somatic mutation classification (Fig. 2b). The MLP model achieved the highest precision (95%) in the held-out test set, and was selected as our predictive model for somatic mutation classification (Fig. 2a). In short, MLP is a class of feedforward artificial neural network. The tuned MLP model has two layers and each layer with 10 and 5 neurons respectively. Learning rate was set as 'invscaling' and solver was set as 'lbfgs'. The 'logistic' activation function was applied in the MLP model.

Validate predicted somatic mutations

We applied the tuned MLP model to predict germline variants/somatic mutations on the mutations derived from the178 benign biopsies without matching germline DNA. In total, out of the 93,653 mutations, 38,210 were predicted to be somatic mutations. To estimate the overall accuracy of predicted somatic mutations, we randomly selected and genotyped three samples for an evaluation study. Three samples were separately LCM dissected. The extracted DNA were genotyped using Infinium Exome-24 Kit. Genotyped probes with GCSCORE larger than 0.15 from the three samples were mapped to hg19 assemblies. Overlapped coordinates were retrieved and the allele frequencies derived from both technologies were compared.

1. Rohan, T.E. & Kandel, R.A. *Breast.* In: E. L. Franco, T. E. Rohan eds. Cancer Precursors: Epidemiology, Detection, and Prevention (Springer-Verlag, New York, 2002).
2. Lakhani, S. R. *et al*. International Agency for Research on Cancer (IARC): WHO Classification of Tumours of the Breast 4 (IARC, Lyon, 2012).
3. Hartmann, L.C.*, et al.* Benign breast disease and the risk of breast cancer. *N Engl J Med* **353**, 229-237 (2005).
4. Wang, Q.*, et al.* Detecting somatic point mutations in cancer genome sequencing data: a comparison of mutation callers. *Genome medicine* **5**, 91 (2013).
5. Wang, K., Li, M. & Hakonarson, H. ANNOVAR: functional annotation of genetic variants from high-throughput sequencing data. *Nucleic Acids Res* **38**, e164 (2010).
6. Martincorena, I.*, et al.* High burden and pervasive positive selection of somatic mutations in normal human skin. *Science* **348**, 880-886 (2015).
7. Flanagan, S.E., Patch, A.-M. & Ellard, S. Using SIFT and PolyPhen to predict loss-of-function and gain-of-function mutations. *Genetic testing and molecular biomarkers* **14**, 533-537 (2010).
8. White, I.R., Royston, P. & Wood, A.M. Multiple imputation using chained equations: issues and guidance for practice. *Statistics in medicine* **30**, 377-399 (2011).
